# Long-term effects of peroxisome proliferator-activated receptor ligand bezafibrate on N-terminal pro-B type natriuretic peptide in patients with advanced functional capacity impairment

**DOI:** 10.1186/1475-2840-8-5

**Published:** 2009-01-28

**Authors:** Koichi Node, Teruo Inoue, Valentin Boyko, Ilan Goldberg, Enrique Z Fisman, Yehuda Adler, Ehud Schwammenthal, Zipora Matas, Solomon Behar, Alexander Tenenbaum

**Affiliations:** 1 Department of Cardiovascular and Renal Medicine, Saga University Faculty of Medicine, Saga, Japan; 2 Israel Society for the Prevention of Hearth Attacks (ISPHA)- the Bezafibrate Infarction Prevention Study Coordinating Center, Neufeld Cardiac Research Institute, Tel-Aviv University, Tel-Aviv, Israel; 3 Cardiac Rehabilitation Institute, the Chaim Sheba Medical Center, Tel-Hashomer, Tel-Aviv University, Tel-Aviv, Israel; 4 Biochemistry Laboratory, Wolfson Medical Center, Holon, Israel, affiliated with the Sackler Faculty of Medicine, Tel-Aviv University, Tel-Aviv, Israel

## Abstract

**Background:**

The effects of pan-peroxisome proliferator-activated receptor (PPAR) ligand bezafibrate on N-terminal pro-B type natriuretic peptide (ProBNP) level in patients with coronary artery disease (CAD) is unknown. The current study aimed to investigate the long-term effects of bezafibrate on ProBNP level in patients with pre-existing CAD and advanced functional capacity impairment.

**Methods:**

Metabolic and inflammatory parameters were analyzed from stored frozen serum samples obtained from 108 patients enrolled in the Bezafibrate Infarction Prevention (BIP) Study. They presented with New York Heart Association (NYHA) functional class III, comprising 58 patients in the bezafibrate group and 50 in the placebo groups, and completed a 2-year prospective, double-blind, placebo-controlled follow-up.

**Results:**

During follow-up ProBNP level did not change significantly in the placebo group, whereas it increased slightly in the bezafibrate group, which was older and with lower baseline ProBNP values. No significant differences between the groups were found for ProBNP levels after 2 year of follow-up. Analysis-of-covariance (ANCOVA) -taking into account age and baseline ProBNP level- showed that bezafibrate was not associated with longitudinal ProBNP changes during the follow-up period (p = 0.3).

**Conclusion:**

Long-term treatment by bezafibrate was not associated with longitudinal ProBNP changes in patients with pre-existing CAD and advanced functional capacity impairment.

## Introduction

Bezafibrate is a pan-peroxisome proliferator-activated receptor (PPAR) ligand with triglyceride-lowering and high density lipoprotein (HDL)-cholesterol raising effects, resulting in decreased systemic availability of fatty acid, diminished fatty acid uptake by muscle, and improvement of insulin sensitizing [1-4]. The Bezafibrate Infarction Prevention (BIP) Study suggested that bezafibrate could prevent secondary cardiovascular events in patients with coronary artery disease (CAD) [5,6]. Although bezafibrate predominantly acts as a PPARα ligand, its effects may be in part due to PPARγ activation [1]. PPARγ activators thiazolidinediones may trigger an aggravation of congestive heart failure [7,9], which counterbalances the cardiovascular potential benefit of these drugs. This appears to be mainly due to fluid retention as a consequence of their insulinomimetic action on the kidney rather than a negative inotropic effect.

Serum N-terminal pro-B type natriuretic peptide (ProBNP) is a strong and independent prognostic marker in patients across the spectrum of heart failure stages. However, the long-term effects of the partial PPARγ agonist bezafibrate on ProBNP level in patients with advanced functional capacity impairment are unknown. Therefore, the current study was designed to investigate the long-term effects of bezafibrate therapy on ProBNP level in patients with pre-existing CAD and advanced functional capacity impairment, using the New York Heart Association (NYHA) class III subgroup of the BIP Study.

## Methods

### Subjects

Metabolic and inflammatory parameters were analyzed from stored frozen serum samples obtained from patients with advanced impaired functional capacity who completed a 2-year of prospective, randomized, double-blind, placebo-controlled Bezafibrate Infarction Prevention (BIP) Study period. The major inclusion and exclusion criteria for the BIP study, as well as the ethical guidelines, have been previously reported [5,10]. In brief, inclusion criteria for men and women comprised: age 45–74 years, history of myocardial infarction no less than 6 months and not more than 5 years prior to enrollment into the study and/or stable CAD confirmed by coronary angiography, and/or radio-nuclear studies or standard exercise tests.

The major exclusion criteria for the BIP study were permanent pacemaker implantation, cerebrovascular disease, chronic hepatic or renal disease, peripheral vascular disease, malignant diseases, estrogen replacement therapy, insulin dependent diabetes mellitus and current use of a lipid modifying drug. The study was a multicenter prospective trial, performed in 18 university-affiliated hospitals. Patients were allocated to receive either 400 mg of bezafibrate retard or placebo once a day, in addition to dietary advice. The follow-up period of the BIP study lasted until May 1998 (mean 6.2 ± 0.8, range 4.7 to 7.6 years).

### Functional status

Functional capacity classes on baseline were evaluated by certified cardiologists, according to the New York Heart Association (NYHA) classification [11], following thorough clinical examinations in the framework of university hospital cardiology departments. Advanced impaired functional capacity was defined as the a presence of NYHA functional class III [12]. There were 134 patients with advanced impaired functional capacity who were included in the BIP study (73 treated by bezafibrate and 61 by placebo); 128 of them survived after a 2-year follow-up (3 deaths were registered both in bezafibrate and placebo group). In 20 patients stored frozen serum samples were missing (12 treated by bezafibrate and 8 by placebo). Therefore, the final study sample comprised all patients with NYHA III and full clinicaly and laboratory data (58 patients on bezafibrate and 50 on placebo).

### Coronary artery disease

The diagnosis of CAD was made in patients with documented myocardial infarction or typical angina pectoris in whom there was also a positive exercise test, evidence of myocardial ischemia revealed by radionuclide studies or at least 60% stenosis of one major coronary artery. Criteria for the diagnosis of MI and anginal syndrome have been previously reported [10]. Briefly, evidence of MI in hospitalized patients (0.5 to 5 years prior to beginning of follow-up) should correspond with *class I*: a) typical symptoms, b) increased serum cardiac enzymes, c) electrocardiographic changes: presence of Q/QS and ST-wave changes according to Minnesota coding, or dynamic changes of ST-segment (depression/elevation) and T waves (increasing/decreasing) on comparing 2 consecutive electrocardiograms; *class II*: b) increased serum cardiac enzymes, c) electrocardiographic changes: presence of Q/QS and ST-wave changes according to Minnesota coding on comparing 2 consecutive electrocardiograms; *class III*: a) typical sympoms, c) electrocardiographic changes: presence of new Q/QS according to Minnesota coding.

Evidence of anginal syndrome (within 2 years preceding commencement of follow-up) should correspond with coronary insufficiency observed at rest or during exertion, manifested by typical pain and dynamic electrocardiographic changes. These included either spontaneous or effort induced ST-segment depression of horizontal or downsloping morphology of ≥1 mm measured at 80 ms from the J point. Reversion to normal or to pre-effort state should be recorded within 1 hour.

### Laboratory methods

Detailed data on laboratory methods were given in previous reports [10,13]. A central laboratory performed all biochemical determinations. For the purpose of the present study, serum samples, which had been taken at baseline from each study participant and stored at -70°C, were thawed. ProBNP determinations were performed within one run, using a Roche Diagnostic ProBNP electrochemiluminescent immunoassay kit on a Elecsys 2010 analyser (Roche Diagnostics, Mannheim, Germany) according to the manufacturer's recommendations. The intra-assay variability of the ProBNP test in our study was 3.9%.

### Determination of additional variables

Criteria for the diagnosis of hypertension have been previously reported. Smoking habits were determined on the basis of self-reporting by the patient during an interview held with a study physician. The homeostatic indexes of insulin resistance (HOMA-IR) were calculated according to the homeostasis model of assessment as follows:

HOMA IR = fasting insulin (μU/ml) × fasting glucose (mmol/l)/22.5 (or fasting glucose in mg/dl/405)

### Statistical analysis

Data were analyzed with SAS software, Version 8.2 (SAS Institute, Cary, NC, USA). Comparisons of dichotomous variables and normally distributed continuous variables were done by the chi-square test and Student's *t*-test respectively. Geometric means (GM) were used for triglycerides, insulin, CRP and ProBNP to take into account their skewed distribution. Non-normally distributed variables were compared by the nonparametric Kruskal-Wallis test, and they were log transformed for further analysis. Pearson's correlation coefficients for the study population as a whole were computed for the association between ProBNP levels and other clinical variables.

Because of their skewed distribution ProBNP was presented as median and interquartile range, GM and 95% confidence interval (CI). Absolute changes (μg/ml) of ProBNP from baseline to 2 year were presented as median and interquartile range and compared using Kruskal-Wallis test. For the assessment of differences in ProBNP values after 2 years between bezafibrate and placebo groups an analysis-of-covariance (ANCOVA) with terms for treatment and baseline values was used based on log-transformed data with adjustment for age.

## Results

### Baseline data and correlations

Patients in the placebo and bezafibrate groups were well balanced in terms of clinical and laboratory baseline characteristics and concomitant medications (Table 1, 2). The study groups were similar regarding gender and the prevalence of the most relevant cardiovascular diseases and risk factors (MI in the past, hypertension, heart failure, peripheral vascular disease, anginal syndrome, chronic obstructive pulmonary disease). Patients in the bezafibrate group were somewhat older (about 2-years difference, p = 0.12). No significant differences between the groups were found for cholesterol and its subfractions, apolipoproteins, blood pressure, heart rate, fasting glucose, triglycerides, fibrinogen, C-reactive protein (CRP), creatinine, fasting insulin and HOMA-IR. Serum baseline concentrations of ProBNP tended to be lower in patients on bezafibrate (p = 0.07). The natural logarithm (ln) of ProBNP at baseline was significantly positively correlated only with age (r = 0.24, p = 0.01). Serum baseline concentrations of ProBNP (pg/ml) in accordance with groups of age are shown in Table 3. Older patients were more frequently on the bezafibrate group (p = 0.04).

**Table 1:** Baseline characteristics of the study population

**Characteristics**	**Bezafibrate** (n = 58)	**Placebo** (n = 50)	**p value**
Age, y	61.1 ± 5.9	59.3 ± 6.2	0.12
Men (%)	53 (91)	46 (92)	0.9
Past myocardial infarction (%)	39 (67)	36 (72)	0.6
Angina (%)	55 (95)	47 (94)	0.9
Hypertension (%)	15 (26)	17 (34)	0.4
Current smokers (%)	7 (12)	6 (12)	0.99
Past smokers (%)	34 (59)	30 (60)	0.9
Body mass index (kg/m2)	27.7 ± 3.8	27.1 ± 3.3	0.4
Systolic blood pressure, mmHg	135 ± 18	133 ± 22	0.5
Diastolic blood pressure, mmHg	81.1 ± 8.1	80.0 ± 10.2	0.4
Glucose, mg/dl	103 ± 19	101 ± 19	0.7
Total cholesterol, mg/dl	211 ± 17	215 ± 18	0.3
HDL-cholesterol, mg/dl	35.4 ± 5.8	35.3 ± 5.1	0.9
LDL-cholesterol, mg/dl	149 ± 15	152 ± 17	0.4
Fibrinogen, mg/dl	348 ± 66	357 ± 70	0.5
Triglycerides, mg/dl	127 (116–140)	135 (124–147)	0.4
CRP (mg/dl)	4.23 (3.40–5.26)	4.66 (3.49–6.23)	0.6
Insulin, μU/ml	4.14 (3.26–5.25)	3.57 (2.70–4.73)	0.4
HOMA-IR	1.02 (0.79–1.31)	0.88 (0.65–1.18)	0.5
ProBNP, pg/ml	154 (118–202)	219 (168–284)	0.07

HDL indicates high density lipoprotein; LDL, low density lipoproteins; CRP, C reactive protein; HOMA-IR, homeostatic index of insulin resistance; proBNP, N-terminal pro-B type natriuretic peptide. Data are mean ± SD, geometric mean (95% confidence interval) or number (%) of patients

**Table 2:** Distribution of cardiovascular drugs among the study patients

**Drugs**	**Bezafibrate** (n = 58)	**Placebo** (n = 50)	**p value**
Beta blockers (%)	29 (50)	26 (52)	0.8
Nitrates (%)	43 (74)	37 (74)	0.99
Calcium antagonists (%)	37 (64)	32 (64)	0.98
Diuretics (%)	6 (10)	10 (20)	0.2
Antiplatelets (%)	39 (67)	36 (72)	0.6
Angiotensin converting enzyme inhibitors (%)	8 (14)	3 (6)	0.2

**Table 3:** Baseline ProBNP (pg/ml) in accordance with age

Groups of age	Patients treated by bezafibrate (%)	ProBNP
I (age≤56, n = 29)	12 (41)	129 (96–172)
II (age 57–62, n = 38)	19 (50)	163 (117–227)
III (age≥63, n = 41)	27 (66)	254 (184–349)

*P for trend*	0.04	0.005

### Effect of Treatment on Changes in ProBNP

Among patients on placebo, baseline ProBNP level was somewhat higher than in patients on bezafibrate, but this difference did not reach statistical significance. No significant differences between the groups were found for ProBNP levels after 2 year follow-up, too (Table 4). Changes in ProBNP from baseline to 2 years of follow-up (bezafibrate vs. placebo) are shown in Table 5. During follow-up ProBNP level did not change significantly in the placebo group, whereas it increased slightly in the bezafibrate group, which was older and with lower baseline ProBNP values. However, ANCOVA, which took into account age and baseline ProBNP level, showed that bezafibrate was not associated with longitudinal ProBNP changes during follow-up (p = 0.3).

**Table 4:** Serum levels of ProBNP (pg/ml) -bezafibrate vs placebo-

	**Bezafibrate** (n = 58)	**Placebo** (n = 50)
Baseline		
Geometric mean (95% CI)	154 (118–202)	219 (168–284)
Median (Interquartile range)	151 (89–335)	215 (101–468)
Mean ± SD	254 ± 295	331 ± 326

P = 0.07 (t-test for ln-transformed values)		
P = 0.11 (Kruskal-Wallis test)		

2-year follow-up		
Geometric mean (95% CI)	206 (157–271)	209 (150–292)
Median (Interquartile range)	230 (100–348)	224 (82–503)
Mean ± SD	374 ± 552	389 ± 442

P = 0.95 (t-test for ln-transformed values)		
P = 0.85 (Kruskal-Wallis test)		

**Table 5:** Changes in the levels of ProBNP from baseline to 2 year follow-up -bezafibrate vs placebo-

	**Bezafibrate** (n = 58)	**Placebo** (n = 50)
Absolute change		
Mean ± SD	120 ± 524	58 ± 307
Median (Interquartile range)	25 (-14–141)	-20 (-102–87)

P = 0.02 (Kruskal-Wallis test, unadjusted)		

Percent change		
Geometric mean (95% CI)	34.1 (8.0–66.3)	-4.3 (-21.8–17.0)
Median (Interquartile range)	21.9 (-9.0–111.8)	-15.8 (-33.1–46.6)

P = 0.3 (ANCOVA, adjusted for age and baseline ProBNP levels)		

P = 0.01 (Kruskal-Wallis test, unadjusted)		

## Discussion

In the present study we have investigated the effects of bezafibrate on ProBNP levels in patients with CAD and advanced functional capacity impairment (NYHA class III). The results indicate a crude trend for serum increase in ProBNP level after 2 years of bezafibrate treatment, compared to placebo. Furthermore, a statistically significant difference between both groups was documented after 2 years treatment for unadjusted ProBNP values. However, age was somewhat higher and baseline ProBNP levels tended to be lower in patients treated by bezafibrate. Taking into account that the baseline natural logarithm of ProBNP was significantly positively correlated with age, we re-assessed intra-group differences by ANCOVA so that the data were adjusted for age and baseline ProBNP level. As a result, it was evident that bezafibrate treatment was not associated with longitudinal ProBNP changes during 2 years follow-up.

In the BIP study, bezafibrate treatment was associated with a nonsignificant risk reduction of secondary coronary events in the entire study population despite a mean of 21% reduction in triglyceride level during a 6.2 year follow up. However, a subgroup of BIP patients with higher triglyceride values (≥200 mg/dl) on bezafibrate treatment exhibited a 43% reduction in recurrent coronary risk compared to the placebo group [5]. In the subgroup of patients with metabolic syndrome, bezafibrate treatment was associated with a reduced risk for onset of myocardial infarction with hazard ratio of 0.71 (95% confidence interval, 0.54–0.95) [14]. In patients with CAD enrolled in the BIP study, bezafibrate treatment significantly attenuated progression of insulin resistance [13] or new onset of type 2 diabetes [15], compared to placebo. In addition, an extended 8.2 year follow-up indicated that treatment with bezafibrate was associated with a significant 17% risk reduction when patients were censored from the analysis upon initiation of therapy with non study lipid lowering drugs [6]. These data support a potential benefit of the treatment with a co-activator of PPARα and PPARγ like bezafibrate for patients with CAD. However, in this analysis when the combined end point of cardiac death or nonfatal MI was analyzed within the prespecified risk subgroups, the benefit of bezafibrate therapy was shown to be most prominent among patients with elevated triglycerides and increased BMI and significantly attenuated among patients with heart failure or ischemic symptoms [6]. Recently, the US Food and Drug Administration required that a "black box" warning for congestive heart failure be placed on the labels of PPARγ ligands of thiazolidinedione group – pioglitazone and rosiglitazone [16]. Thiazolidinediones, as a class, are well known to increase fluid retention through unknown mechanisms, which appear to be the primary contributor to the increased risk of congestive heart failure with thiazolidinediones [7-9]. In addition to PPARγ, in mouse models of cardiac hypertrophy and heart failure, the activation of PPARα has also been shown to cause noxious effects on the development of the ventricular function [17,19]. Thus, theoretically caution should be ought in the use of bezafibrate in patients with advanced heart failure.

Across the spectrum of heart failure stages, assessment of ProBNP levels at a single time point in stable outpatient settings provides a powerful independent prediction of mortality and new events. Serial testing provides incremental prognostic information: a decrease in levels at follow-up predicts fewer heart failure hospitalizations or deaths, and an increase in levels predicts a greater likelihood of these adverse outcomes [19-21]. Contrary to bioactive BNP, biologic activity is absent in ProBNP and its measurement provides us stable data. Given that ProBNP levels decrease in response to the addition of therapies with proven benefit for heart failure, it may be expected that targeting a given therapy to decrease ProBNP levels may reduce adverse clinical outcomes. ProBNP secretion is nonlinear, and when log-transformed peptide levels are assessed in stable patients, as done in the current study, these levels appear constant with a variability less than 10% [22,23]. The clinical interpretation is often confounded by factors that influence the plasma levels of ProBNP. For instance, an inverse relation between body mass index and ProBNP has been observed in patients with [24] and without heart failure [25,26]. In the current study, the natural logarithm of ProBNP at baseline was positively correlated only with age but not with body mass index. Similarly, renal dysfunction may also influence the plasma levels of ProBNP, further complicating the interpretation of the levels. However, it is logical to assume that elevated ProBNP values in patients with combined chronic heart failure and chronic kidney disease have a higher risk for adverse outcomes [27]. The results of the current study, which indicates no adverse effects of active treatment on longitudinal ProBNP changes, did not support a special restriction for bezafibrate use in patients even with advanced functional capacity impairment such as a NYHA class III.

### Study limitations

The current study included relatively few subjects to provide definitive explanations regarding the intergroup differences in the observed ProBNP level changes at baseline and at end of follow-up. The analyses were performed retrospectively using stored frozen serum samples obtained from patients in a subgroup of BIP Study. In addition, any other data for cardiac function such as echocardiographic parameters were not available. Future larger-scale controlled clinical trials targeting cardiac function evaluations including ProBNP level as a primary endpoint are required to establish the safety of bezafibrate treatment in CAD patients with advanced functional capacity impairment.

## Conclusion

Long-term bezafibrate treatment was not associated with longitudinal ProBNP changes in pre-existing CAD patients with advanced functional capacity impairment.

## Competing interests

The authors declare that they have no competing interests.

## Authors' contributions

KN conceived the study; TI and AT drafted the manuscript; EZF, KN, TI, YA, SB, ZM and AT were involved in the study design, coordination, laboratory measurements and data acquisition; AT, VB, ZM and SB studied and matched the records from the BIP study; EZF, IG, ES, and ZM interpreted the results and VB performed the statistical analysis of the data presented; EZF, KN, SB, ES, ZM and IG critically reviewed the study for important intellectual content. All authors approved the final version of the manuscript.
